# A Rollercoaster of Grades Versus Growth in the Clerkship Year: A Phenomenological Study of Medical Student Experience with Competency Development

**DOI:** 10.5334/pme.1564

**Published:** 2024-11-25

**Authors:** Matthew Kelleher, Benjamin Kinnear, Danielle Weber, Abigail Martini, Sally A. Santen, Pamela Baker, Laurah Turner, Eric Warm, Melissa Klein, Daniel Schumacher

**Affiliations:** 1 University of Cincinnati College of Medicine, Cincinnati Children’s Hospital Medical Center, Cincinnati, Ohio, US; 2 University of Cincinnati College of Medicine, Cincinnati, Ohio, US; 3 Cincinnati Childrens Hospital Medical Center, Ohio, US; 4 Department of Medical Education, University of Cincinnati College of Medicine, Cincinnati, Ohio, US

## Abstract

**Purpose:**

As competency-based medical education (CBME) continues to advance in undergraduate medical education, students are expected to simultaneously pursue their competency development while also discriminating themselves for residency selection. During the foundational clerkship year, it is important to understand how these seemingly competing goals are navigated.

**Methods:**

In this phenomenological qualitative study, the authors describe the experience of 15 clerkship students taking part in a pilot pathway seeking to implement CBME principles. These students experienced the same clerkship curriculum and requirements with additional CBME components such as coaching, an entrustment committee to review their data, a dashboard to visualize their assessment data in real-time, and meeting as a community of practice.

**Results:**

Students shared their experiences with growth during the clerkship year. They conveyed the importance of learning from mistakes, but pushing past their discomfort with imperfect performance was a challenge when they feel pressure to perform well for grades. This tension led to significant effort spent on impression management while also trying to identify their role, clarify expectations, and learn to navigate feedback.

**Conclusions:**

Tension exists in the clinical environment for clerkship students between an orientation that focuses on maximizing grades versus maximizing growth. The former defined an era of medical education that is fading, while the latter offers a new vision for the future. The threats posed by continuing to grade and rank students seems incompatible with goals of implementing CBME.

## Introduction

The education system immerses medical students in an environment that simultaneously promotes the development of competence, while also providing incentives to conceal weaknesses and stand out among their peers [1]. It is unclear how this tension impacts student competency development during the clinical years. Previous work has focused largely on assessment of competence including best practices for assessment using the Core Entrustable Professional Activities for Entering Residency (CEPAERs) [2], and work-place based assessment (WBA) [3], seeking validity evidence for entrustment decisions [4], and making summative decisions regarding competency development [5].

While important in defining future efforts, this previous work has rarely sought meaningful understanding of the student perspective of competency development, particularly the process and factors influencing it. Complicating this, efforts to implement competency-based medical education (CBME) face obstacles that may influence students’ experiences with competency development, such as the competitive demands of residency selection [6,7]. The residency selection process in the United States creates pressure on medical schools to discriminate between and normatively compare students, which is incongruent with core principles of CBME that include criterion-referencing and formative development [8,9,10]. This often results in layering components of CBME onto curricula where normative-based assessments, like tiered grading, awards, and class rank, continue to exist.

The clerkship year, marking students’ first immersive experience in the authentic healthcare workplace, is a formative transition from the classroom to clinical settings. It exposes students to different clinical disciplines and their norms, socializing them into the profession, promoting identity formation and guiding career path exploration [11,12]. Educational uncertainty abounds as students attempt to learn new skills while navigating new healthcare environments [13]. Students are particularly susceptible to peripheral participation. Supervising teams and workplace activities can be challenges to making their participation and socialization feel more legitimate [14]. Understanding students’ experiences managing competing priorities and how they navigate their own growth and competency development within that complex setting is crucial. This understanding can inform the creation and refinement of optimal clinical learning environments. Therefore, it is not enough to merely assess competencies; we must also understand how students engage in the process of competency development from their perspective. In this study, we explored students’ experiences and perceptions of their own growth and competency development as they navigate the clerkship year while part of a CBME pilot.

## Methods

### Design

We employed hermeneutic phenomenology [15,16], a qualitative approach that attempts to describe a phenomenon within participants’ lived experiences. We explored students’ experiences with and conceptualizations of competency development in the unique context of the clinical learning environment and clerkship structure. Competence was defined to the students as the knowledge, skills, and attitudes necessary to care for patients. The students interviewed were part of a CBME pilot pathway, which encouraged them to frequently reflect on their own growth and development. As such this group offered valuable insight into this research question. We engaged in this study from a social constructionist worldview. This research was reviewed and determined to be exempt by the University of Cincinnati Institutional Review Board.

### Pathway and participants

In Spring 2021, rising clerkship students at the University of Cincinnati College of Medicine (UCCOM) applied to join a CBME pilot pathway. Four faculty members reviewed blinded applications to select motivated students whose intentions aligned with the pathway’s aims: 1) promote and support students using assessment data to guide growth, and 2) develop the ability to make competency-based decisions about student development during the clerkship year. Fifteen students, out of thirty-nine applications, were selected based on the feasibility of pathway size. Seven students identified as female, 7 male, and 1 as non-binary. Three students identified as Black, 1 American Indian, and 2 Asian. One student was in the MD/PhD program.

Pathway students’ clerkship schedules and curricula did not change; however, several additions were made for students in the pilot pathway. First, each student was paired with a faculty coach to help them reflect during the year. Coaches were faculty members from various specialties trained in the aims of the pathway, coaching theory and techniques, and the master adaptive learner framework [17,18]. Coaches were expected to meet with students every six to eight weeks.

Second, the pathway was focused on competence assessment, so students had a unique QR code that allowed them to ask for narrative feedback from any healthcare professional they worked with and view it on their own dashboard. Pathway students had unique fields for input from different healthcare professionals and were encouraged to actively seek feedback. QR assessments were completely narrative-based and the prompts asked assessors to describe components of the Association of American Medical Colleges (AAMC) Core Entrustable Professional Activities for Entering Residency (CEPAERs) they trusted the student to perform.

Third, a competency committee was formed that made entrustment decisions using the AAMC CEPAERs. Students in the pilot were introduced to these during an introductory meeting prior to the start of clerkship year. For the traditional students, EPAs are not used as the organizing competency framework and there is no competency committee to review students’ assessment data across the clerkships at UCCOM.

Fourth, every six to eight weeks pathway students met as a community of practice to share their experience and integration into the clinical learning environment. These sessions were informal, and the pathway director was present to help facilitate the conversation. At the start of clerkship year, the students also received three narrated PowerPoints on learning during the clerkship year, the role of a coach, and the logistics of the pilot pathway.

### Data collection

We interviewed all 15 pathway students between October and December 2021, approximately halfway through their clerkship year. A research coordinator (AM) who was not involved in student assessment performed all interviews. The semi-structured interview guide (appendix) aimed to understand how pathway students think about their performance, measure their progress, develop competence (including accelerators and barriers), and gather feedback in the clinical learning environment. We developed the interview guide and made changes after a pilot interview with a traditional clerkship student. We transcribed interviews, which ranged in length from 17 to 58 minutes (average 32 minutes).

### Data analysis

Consistent with hermeneutic phenomenology, we sought to describe how competency develops within the lived experience of students, including the contextual forces that shape it [15,16]. Data collection and analysis were iterative, as detailed below. After de-identifying transcripts, four authors (BK, MEK, AM, DW) developed first-order constructs (meaningful bits of data that retain the words of the participants as much as possible) for four initial interviews. After discussing as a group, the first author (MEK) compiled the first-order constructs of each co-author, which were reviewed with the entire author team to gather feedback. Detailed notes were taken to further elaborate these discussions using the research teams’ theoretical knowledge and personal experiences. The smaller team of four authors continued to review new transcripts in small groupings to further develop first and second-order constructs. An example of the relationship between first and second-order constructs might be moving from a specific example of how a student measured their progress to a second-order construct such as internal and external factors to gauge growth. Group discussions among these authors continued after interviews 4, 6, 9, 12, and 15. Once established, the first author (MEK) presented second-order constructs to the larger author team to clarify meaning and attempt to incorporate these into themes based upon the full research team’s experience. The first author (MEK) then engaged in iterative cycles describing how individual themes related to the whole phenomena of student competency development with input from the smaller team of co-authors that participated in primary analysis. As analysis neared completion, the first author returned to review the 15 transcripts to further elaborate these themes and finally compose a narrative that includes four themes and describes how students experience competency development in the clerkship year.

### Reflexivity

Given that hermeneutic phenomenology includes the researchers’ lived experiences, we reflected on the experiences and perspectives that were integral to our data interpretations. BK, MEK, and DW are all practicing internists and pediatricians who are engaged in medical education research and have their own personal experience with the clerkship year. Two authors (MEK, DW) co-direct a course for first- and second-year medical students to learn the fundamental skills of doctoring in a simulation setting. All of the authors are active in CBME initiatives and assessment of trainees. The larger author group includes representation from graduate medical education (GME) and undergraduate medical education (UME) with residency program directors, associate and assistant deans, emergency medicine, general pediatrics, and medical education research scientists.

## Results

Students described their competency development as apparent when “reflecting on where I used to be.” (Student #5 (S5)) However, day-to-day was more challenging to know if they were growing, leading to doubts about their own abilities. Their real-time uncertainty seems complicated by constantly surveying the clinical learning environment to determine if “it’s okay to make mistakes” (S12) or whether there is “big pressure to perform.” (S11) We describe four themes to illustrate pathway students’ experiences with competency development. These themes focus on students believing that stepping outside of their comfort zone is critical for growth, but this is not easy when they feel external pressure to perform well. As a result, much effort is spent worrying about impression management while they try to learn their role and navigate feedback in a learning environment that often feels like a rollercoaster.

### Pushing past discomfort is critical to growth

Students believed their growth and development requires trial and error, making mistakes, opportunities to practice, and potentially fail. Students described how “you have to get over yourself” (S2) and “try things, fail, readjust, and retry” (S5) since “failing is part of growth.” (S9) However, students did recognize that while mistakes are invaluable for growth, they must “not put patient [safety] at risk.” (S12) Ideally, failing safely leads to immediate feedback and/or opportunities to intentionally reflect and target specific deficiencies. For example, one student stated, “I’m developing because I am using my time wisely to focus on the skills that I’m not as strong in.” (S6) Unfortunately, opportunities to practice and fail are not always available for many reasons (detailed below) and students reported this “hurts your personal growth.” (S2)

While all students recognized the importance of practice and mistakes, few enjoyed it. Many spoke of their own struggle to overcome the discomfort of being wrong, including not taking things personally and reframing it as an opportunity to learn and retry. One student noted the importance of “having a growth mindset and not really putting myself down if I am doing something wrong because it’s part of the process.” (S14) Students described their own responsibility, sometimes having to advocate for their own skill development by asking specifically to see patients, practice a skill, or say yes to opportunities despite major internal doubts. They relayed a choice created during the clerkship year when they are in “no man’s land between observation and participation” (S9) and the importance of “breaking the barrier of comfortability and doing something” (S14) and thus the “role that being uncomfortable can play in growth.” (S14)

### External pressure from grades leads to impression management

Students were most willing to embrace discomfort if they felt educationally safe. This often meant they felt free to “share and ask questions and to suggest things” (S11) without it negatively impacting their grades. In these situations, students described an ideal environment for their growth and competency development. Situations where students do not feel safe being wrong were described as the “most harmful learning environment.” (S5) They described a “different way of thinking and acting” (S14) when their focus was on grades versus growth. One student described how difficult it is “going through so much schooling and being so focused on grades to transition to thinking about development as not just the numbers associated with your grades.” (S6) Students felt grading “doesn’t always align with the definition of competence” (S12) and relayed that you can “pass your clerkship[s] and do well and that may [not] translate to being the best resident.” (S4)

Many reasons were given for why grades create pressure and hinder growth, including how subjective and stylistic grades feel, discordance with other feedback students receive, interpersonal dynamics such as a bias, and a lack of direct observation or clear criteria to inform grades. For these reasons, calculated grades from workplace-based assessments were viewed as “frustrating”, (S6) “not helpful,” (S2) and a “dice roll” (S9). This ultimately undermines growth in their view. One participant summarized this sentiment by noting that if they focus on grades too much they would “be miserable.” (S3)

Despite a desire to focus on growth and development, many students felt a constant pull to perform since they are being graded, making it hard “to be authentic”. (S15) One student noted: “people aren’t really themselves; they aren’t asking questions for the sake of learning.” (S1) Putting on this performance was described as pressure, stressful, and a hindrance to growth. Students described a significant amount of cognitive energy spent trying to “be what they think preceptors want them to be.” (S14) This sometimes even resulted in “weird social interactions” (S5) where they are monitoring if the climate is right to ask for feedback, if they could or should ask questions (but not too many), and how to look curious, eager, and smart while hiding their weaknesses and never “slowing the team down.” (S12) One student summarized it as “basically pleasing whoever is in charge.” (S15) All of this mental and emotional energy spent on impression management was identified as a deterrent to individual growth since it limited opportunities to practice and potentially learn. Students acknowledged this tradeoff between performing for grades and focusing on growth, sometimes even lamenting the fact that “I will not be as well-rounded” (S9) with less energy spent on preparing to be a physician.

### Identifying a role and expectations is a “rollercoaster”

Energy spent constantly trying to adapt to supervisors was also compounded by switching from one clerkship to another, which felt like a “gauntlet or rollercoaster.” (S9) Adjusting often to new teams, new styles, and new clerkships made it difficult to “carve out their role” (S2) and at times led to “trying to stay out of the way.” (S2) To overcome this tendency, students felt they needed to “advocate for [their] own learning needs” (S1) or intentionally ask about expectations for them. This was met with mixed results as some attendings were said to have “low” (S4) or “no expectations” (S1) for clerkship students.

Given murky expectations, determining if their performance was on target was a challenge for students. Some were using their own goals as a target while others were comparing or modeling themselves after fourth-year medical students and other peers. Students recognized that self-assessment is insufficient, and one student related this to learning good vibrato: “It takes a musician to say, you aren’t quite there yet, keep working on this.” (S7) Another student described their clerkship year as “mindlessly bumping into things” (S3) and “performing randomly” (S3) when feedback was not available. Despite the importance of feedback, students remarked that it was an “imperfect judge,” (S12) and they “rarely get feedback if [they] don’t ask for it.” (S1) Therefore, students shared how they have learned to navigate the clinical learning environment to guide their growth, noting that “you can develop competence unintentionally, but I think you can develop it faster if you’re intentional about it.” (S14).

### Learning to navigate feedback while trying to gauge competence

Since gauging their own progress can be a challenge, many students described “fear that there is something [they are] missing” (S5) and simply being unaware of their own weaknesses. This led many students to describe “competency development [as] a feeling.” (S5) When they *feel* comfortable and confident, they believe growth has occurred. Related to this, “how much they are trusted to do” (S3) by the residents is another indicator, as more autonomy and more responsibility were understood as signs of growth. Similarly, they also believed competence was reflected by how many additions or modifications were made to their work. For example, “writ[ing] a note and there’s very little edits” (S2), “how many additional questions did the resident need to ask” (S1) after a patient interview, or whether they “do a physical exam and have my findings be validated” (S3) by others.

While all students described receiving feedback, this seemed insufficient to gauge their competency development due to the generic or vague feedback often received. However, some students felt you could learn to get “better ask[ing] for feedback” (S13) and found value asking before performing a skill to prime the assessor for direct observation. Others described asking for feedback immediately after an oral presentation, physical exam, or procedure while their performance was still “fresh in your mind.” (S3) Students learned to acknowledge general weaknesses when asking for feedback to focus the preceptor on more targeted areas. For example, “I know medical knowledge is something I’m going to need to work on, do you have any other suggestions on how I can improve X or Y?” (S4) Related to this, students also learned to “critically appraise feedback” (S3) over time, understanding that it is acceptable to disagree. Students felt feedback was most useful when they were able to reflect on patterns from different sources in different contexts or clerkships. Because of this they saw value in having the feedback written down, and some students referenced their own process to keep track of verbal feedback.

Students expressed that assessors’ inability or unwillingness to provide constructive feedback was a major limitation since that feedback was necessary for growth. Recognizing this limitation, students tried to “set a tone” (S15) or “create a mood” (S1) to show they wanted constructive feedback before supervisors were willing to share specific details for improvement. Students appreciated when the stakes for feedback were low and disconnected from a grade because it allowed them to “focus on the negative” (S9) but they still acknowledged a need for “growth mindset” (S12) to not take it personally.

## Discussion

In this study, we sought to understand how students in a CBME pilot pathway experience competency development during the clerkship year, revealing a complex interplay between internal drive for growth and stress created from external pressures. Students struggle to gauge their own competency development. They described the need to try and sometimes fail to actually grow. However, when these opportunities were available, there was apprehension related to how those mistakes may impact their grade. This led to significant cognitive dissonance as they try to manage impressions and satisfy supervisors. The results of this study seem to align and advance the conversation of others indicating that most students believe their development is hindered during their clerkship year by grading systems that do not encourage the kind of behavior that ultimately leads to growth [1,5,19,20]. These results argue that CBME and grades cannot coexist, a clear warning for many who wish to have both.

### Implications for Medical Education Leaders

There is very little evidence that clerkship grades predict future clinical performance or represent true differences in clinical performance [21,22,23,24,25,26]. Adding this to our findings and the findings of others, we believe it is simply not defensible to continue grading and ranking students during the clerkship year. As CBME efforts in undergraduate medical education (UME) grow, there is a building tension on students that cannot be ignored. Students are dealing with competing priorities, illustrated in our findings, torn between focusing on maximizing grades or growth. Even in the best circumstances for CBME implementation, as long as UME continues to also try to meet the demands of residency selection by ranking and comparing students, learners will continue to struggle with this tension, limiting their growth potential [9,10,27,28].

Beyond maximizing growth potential, removing normative grades is also one of the few interventions shown to reduce burnout, anxiety, and stress in students [29,30,31]. It seems plausible that such consequences are at least in part due to students trying to meet the competing demands of comparison. They seem to understand all too well that it is not good enough to reach performance metrics; they need to reach them without mistakes and while standing out from their peers.

Bearing the evidence, the time has arrived to make the transition away from normative grades to growth. Currently, AAMC reports only 13% of schools have a clerkship year without grades [32]. But obstacles exist. Simply removing normative grades will not replace the need to improve programmatic assessment, mitigate bias, clarify outcomes, better define the roles of clerkship students, and maximize direct observation in the clinical learning environment. Achieving these goals in a grade-free world are discussions that many students would likely welcome, but progress cannot begin if normative grades continue to cloud the conversation. But, some students, especially those who benefit from grades via normative comparisons that put them on top, may resist their removal. However, if we know these grades are biased and flawed and interfere with efforts to produce the best physicians we can, should we allow this privilege to prevail? We think not.

### Implications for Learners

Our findings suggest the clerkship year can be an internal battle for students. Faced with many sources of uncertainty, they need opportunities to learn how to tolerate this without the fear of grades also looming [13,33,34]. Some have also shown how cognitive waste spent on impression management leaves trainees with an emotional burden and often hinders growth [35,36,37]. While this tension could be diminished by removing grades, it is unlikely to go away completely. The tightrope between expressing vulnerability and appearing credible is a lifelong pursuit that is particularly challenging when students’ participation feels peripheral, and they are frequently encountering new teams [14,38]. Helping students become more aware of their cognitive and behavioral responses may help them manage all of the uncertainty they face in the clerkship year [33,39]. Since students are often seeing and doing things for the first time, many of them describe a growth mindset as a necessity [40]. This approach enables them to improve how they seek feedback, embrace challenging opportunities, and remain open with themselves and others when they do not understand something. Students should be intentionally taught and prepared for this, since embracing a clear understanding of growth mindset might be the most important outcome of the entire clerkship year [41,42,43,44].

Beyond a growth mindset, many students in our study commented how easy it is to continue blending in during the clerkship year. Expectations are variable and feedback is infrequent if it is not sought. Many students described how important it was to push past discomfort and how less than ideal performance often leads to the best opportunities for learning and gauging their own growth. This was remarkably similar to how students described their cognitive, emotional, and behavioral responses to uncertainty [33]. As a result, there was a common belief amongst students that their competency development was best understood as a feeling. Students seemed to comprehend that tangible evidence to support their competency development was often insufficient, but despite this, they could feel when growth was happening. This feeling is worth exploring further as many warn of the discrepancies between confidence and competence [45,46,47]. Some have suggested the importance of “informed self-assessment,” but this likely requires more meaningful assessment data on performance and potentially even a coaching relationship to help that learner reflect [48]. Unfortunately, valuable reflections on assessment data feels impossible while that same data is also being used to calculate a grade.

### Implications for Schools

One question that resulted from hearing how students experience competency development was: What is the purpose of the clerkship year? Our pathway students often found it challenging to carve out a role, leading one to wonder if the clerkship year is really about career exploration and socialization into the profession of medicine. Despite this, performance during the clerkship year remains a significant factor in how residency programs sort applicants. It is challenging to reconcile goals of career exploration and socialization into a profession, while also collecting high stakes assessments on performance of many tasks that students perform for the first time. This is another reason to remove high stakes normative grades from observational assessments during the clerkship year and doing so would allow schools to be clearer about the purpose and expectations for both students and faculty during this critical year. By continuing to focus on normative comparisons, many schools are effectively saying that the clerkship year’s purpose is sorting and competition.

Students also described significant differences in the learning environment between different supervisors. Clinician engagement is critical in legitimizing student participation and the development of skills and knowledge [14]. It is unclear what should be the acceptable variability within one single clerkship. Should our learners be expected to adapt to many different styles and ranges of expectations? Many students are spending excessive amounts of cognitive energy trying to guess or adapt to different faculty expectations and styles. Faculty development is always a challenge and easy to blame but there seems to be an opportunity to improve the consistency between faculty. In doing so, we can potentially free up cognitive space for students to focus on the knowledge, skills, and attitudes we hope they develop [49,50].

## Limitations

The experience and description of competency development is a complex phenomenon. This study was not designed to evaluate the success or failures of the CBME pilot or interventions. The depth of understanding and insights students shared, however, was certainly impacted by their involvement and constant reflection on CBME principles during the previous six months. Thus, while our data provides a rich description of competency development from the student perspective, it is likely incomplete and may not be representative of the experience of all medical students or those at other institutions. The pressure that these students feel may not be the same at other institutions that have different resources, demographics, and prestige. The approach these students shared to navigate growth in their clerkship year might also be different at other institutions.

## Conclusion

The findings of this study offer yet another call for UME to move toward CBME by focusing on the development of our students over the continued focus on grading them for the purposes of selection. The clerkship year serves multiple purposes, and many opportunities exist to improve students’ ability to navigate growth during this formative year.

## Additional File

The additional file for this article can be found as follows:

10.5334/pme.1564.s1 Appendix. Semi-structured interview guide.
