# Supplementary material for: A Rollercoaster of Grades Versus Growth in the Clerkship Year: A Phenomenological Study of Medical Student Experience with Competency Development
Source: Perspect Med Educ. 2024 Nov 25;13(1):592–601. doi: 10.5334/pme.1564 (PMC11606390; doi:10.5334/pme.1564)
Supplement: Appendix. — Semi-structured interview guide. [file pme-13-1-1564-s1.pdf]

Semi-Structured Interview Questions

Date, Name, Year in school

What clerkship are you currently rotating on and which clerkship have you completed so far?

1) Can you share with us an example or a story that might describe your performance (can be good or bad or both) so far during this clinical clerkship year?
2) How do you know if your performance is on target?
3) If we define competence as the knowledge, skills, and attitudes necessary to care for patients then how do you think <u>a student</u> should gauge their competency development during clinical clerkships?
    a. What evidence would you point towards to describe your own competence?
4) How do you think <u>you</u> develop competence?
    a. Do you think it is possible for some students to develop faster than others?
    b. If yes, what recommendations would you give to help a student develop faster?
    c. If no, can you tell us why not?
5) What aspects of the clinical learning environment (define CLE) help and/or hinder your own personal growth and development of competence?
6) Can you share an example of an experience that changed (or emphasized) your approach to development in the clerkship year?
    a. Can you share an example of feedback you received that changed your approach?
    b. Can you share an example of an assessment of your performance you received (i.e. preceptor evaluation, OSCE, etc.) that changed your approach?
7) What are the differences between feedback you initiate (ask for) and feedback provided to you that you did not seek? (if answer appears vague, can ask for examples)
